# The Prevalence of Caregiving among Young People

**DOI:** 10.3390/ijerph21050621

**Published:** 2024-05-14

**Authors:** Lawrence T. Lam, Mary K. Lam

**Affiliations:** 1 Faculty of Medicine, Macau University of Science and Technology, Macau SAR 999078, China; 2 Faculty of Health, University of Technology Sydney, Sydney, NSW 2007, Australia; 3 School of Health and Biomedical Science, RMIT University, Melbourne, VIC 3000, Australia; mary.lam@rmit.edu.au

**Keywords:** caregiving, young caregivers, prevalence, systematic review, meta-analysis

## Abstract

With the increasing number of people with chronic diseases and disabilities, the number of family members as caregivers have also been growing. Despite the attention paid to caregiving in recent years, little is known about caregiving among young people, particularly its global prevalence. The lack of information has important implications for health policy and management, resulting in the inability to form appropriate evidence-based policies and managerial decision making. This study aims to derive an estimate of the prevalence of caregiving among young people through a systematic review of the current literature. The results of this study revealed a prevalence of caregiving among younger adolescents of between 1.1% (1.06–1.14%) and 12.0% (11.02–12.98%). However, the assessment of caregiving varies across studies, and all were conducted in developed countries. These results provide information on the burden of caregiving in young people and reveal the lack of global information, calling for more research on and attention to this specific population.

## 1. Introduction

It has long been recognised that the number of people living with chronic diseases and conditions has been increasing rapidly [1]. As a result, the number of patients requiring medical attention and care has also increased significantly. This has been reflected in the increase of individuals providing informal care to relatives and loved ones with chronic illnesses or conditions. The World Health Organization defines a caregiver as “a person who provides support and assistance, formal or informal, with various activities to persons with disabilities or long-term conditions, or individuals who are elderly. This person may provide emotional or financial support, as well as hands-on help with different tasks” [2]. By this definition, there is no distinction between the quantity and the extent of care. While most caregivers are adults, a proportion are young people, even as young as children [2]. Young caregivers or carers have been defined as children and young people under the age of 18 years who provide care to a family member with various health conditions [3]. These conditions may include chronic illnesses, disability, mental problems, and other conditions, such as substance abuse and old age frailty [3].

Caregiving may have detrimental effects on the mental and physical health, as well as the economic and financial well-being, of caregivers. It was shown that caregiving is associated with an increased risk of psychological stress, anxiety and depression, physical ill health, and impaired social and family life [4,5,6,7,8]. Providing long-term care for chronically ill family members or significant others at home also increases the risk of financial difficulties for caregivers [8]. Caregivers who were employed have reported missing work and taking personal days off, early retirement, and reduced paid working hours to provide care [9,10]. Caregivers have also reported feeling tired, isolated in a mostly unsupported role, reduced quality of life, increased burden, more physical morbidities, and even suicidal ideation [8,11,12,13,14]. They also reported lower levels of subjective physical health and less self-efficacy [15]. While there has been a growing number of studies on the impact of caregiving on adults, little is known about the effect of caregiving on young people. Research on caregiving among young people suggests both positive benefits as well negative consequences of a caregiving role [16,17,18,19,20,21,22]. Caregiving could promote positive perceptions of self-identity and self-worth and create a sense of belonging in young caregivers [16]. Adolescents who had been involved in caring at home reported having an increased self-esteem and feelings of interpersonal competence [17]. Caregiving could provide young people with a meaningful connection to other people and thus help with developing a positive self-identity [17]. However, the majority of studies have reported negative impacts of caring on young caregivers, including negative emotional well-being, physical ill health, lowering of educational achievement, and psychosocial maladjustments [18,19,20,21,22,23,24]. In a U.K. study, it was reported that 22% of young caregivers aged between 5 and 10 years missed school or experienced educational difficulties as a result of their caring activities [22]. The results from two larger studies suggested that child caregivers experience more anxiety and antisocial behaviour than noncaregivers [25,26].

Due to the aforementioned effects of caregiving on young caregivers’ socioemotional health, mental health, and well-being, as well as the fact these are children still going through growth and development, these negative effects are detrimental to their physical, socioemotional, and mental health. These effects also affect their ongoing development into adulthood and may affect their chance of accessing higher education as well as employment opportunities in the future [27,28]. To date, information on the magnitude of young people providing care or the prevalence of caregiving globally and the burden of caregiving on these young people is scarce and fragmented [29]. Aldridge noted that obtaining an accurate estimate of the prevalence of young people’s caregiving is not an easy task [29]. Two main reasons for this were proposed: in the past, there was a lack of clear definition of the age of young caregivers until Becker’s work in 2000 [30], and it was challenging to define the nature, frequency, and duration of the care provided [29].

There are many implications of the lack of knowledge of the prevalence of young caregivers. On the practical level, it limits the provision of services to support this specific group of young people. At the health policy and management level, the lack of information imposes a severe restriction on the formulation of appropriate evidence-based policies as well as managerial decision making. Hence, it is prudent to gain a better understanding of the magnitude of young people who assume a caregiver’s role.

## 2. Methods

### 2.1. Search Strategies

The PRISMA guidelines for systematic reviews and meta-analyses were applied to perform a search of the literature through electronic databases using a systematic strategy [31]. Major medical, health, psychological, and educational literature databases including Cumulative Index to Nursing and Allied Health Literature (CINAHL), PsycINFO, Medline, Education Resources Information Centre (ERIC), Education Research Complete, Academic Search Complete, Business Source Complete, and ProQuest were employed to search for relevant articles. The keywords used for the systematic search were (“caregiver” or “informal caregiver” or “unpaid caregiver”) AND (“young” or “child” or “children” or “adolescents” or “young adults”) AND (“prevalence”) AND (“chronic illness” or “chronic condition”). Included in the search were articles published as reviews to identify relevant studies. However, these review articles were not included in this systematic review. Limitations were imposed on the search of publications to those the English language only, including prevalence data on people aged under 18 years and restricting the publication years to include studies published between January 2010 and December 2023. This criterion was used to reflect the rapid increase in people developing and living with chronic diseases in the past decade and to accurately reflect the prevalence of caregiving in young people. Upon completion of the search on the electronic database, the titles and abstracts of the identified articles or reports were assessed for their suitability to be included in this review. After assessing the titles and abstracts, the full texts of the articles or reports deemed suitable were retrieved for inclusion in the review. Other potential articles that could have contained information on the prevalence of young caregivers were also sought through the reference lists of the included articles and other grey literature, such as government reports.

### 2.2. Selection of Articles

The following selection criteria were applied for suitable articles: (1) peer-reviewed cross-sectional or cohort studies or published reports; (2) the prevalence of young caregivers (under 18 years) was provided or could be calculated from available data; (3) articles or reports published between January 2010 and December 2023 with data obtained within the study period; and (4) articles or reports published in the English language.

All articles identified in the search were imported to the Covidence online platform [32]. Titles and abstracts of the captured articles or reports were screened for eligibility using the inclusion criteria. Articles not meeting the eligibility criteria were excluded. Articles or reports meeting eligibility criteria were identified, and the full texts of these articles or reports were obtained. The contents of these articles or reports were independently assessed against inclusion criteria by two researchers (LL and ML). Any inconsistencies were discussed until a consensus was reached.

### 2.3. Assessment of the Study Quality

The main aim of this systematic review and meta-analysis was to estimate the prevalence of caregiving among children and young adolescents under the age of 18 years only; it was not meant to explore any other analytical aims. As a result, most of the methodological requirements for good quality analytical studies did not apply to the current study, except for the sample and the sampling approach. As such, the quality of the included studies was assessed descriptively instead of employing a structured assessment using a standardised approach, such as the Critical Appraisal tools for use in JBI Systematic Reviews [33].

### 2.4. Information Extraction and Analysis

Information was extracted from the selected articles and tabulated for further analysis. Information extracted from selected studies included authors, year of the study, location of the study, study design, the sample population, prevalence estimates stratified by age group as <15 years and 15–17 years (where the information was available), and other information or remarks relevant to the study. The 95% confidence intervals (95% CIs) of the reported prevalence were also calculated using the available information provided in each article or report.

## 3. Results

**Figure 1:**
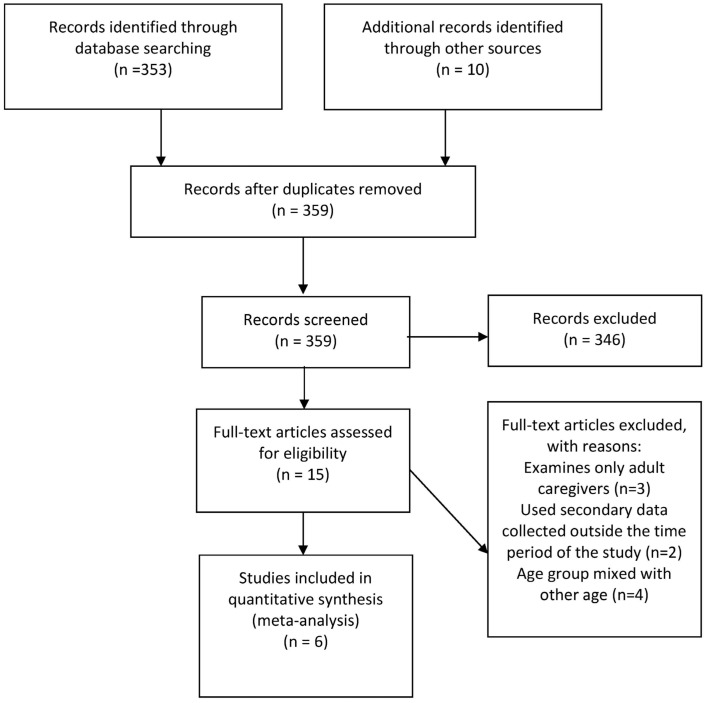
PRISMA flowchart of the search for peer-reviewed journal articles and other reports.

Using the described strategy, an extensive search of the literature was conducted, resulting in 359 articles related to young caregivers in the English language being identified after duplicates were removed. After assessing the title and abstract of these articles or reports, 15 were found to potentially meet the criteria for this review [28,34,35,36,37,38,39,40,41,42,43,44,45,46,47]. The selection of these 15 articles was based on the criteria stated in the Methods Section. A closer examination of the full text led to only six studies, including four peer-reviewed journal articles and two government reports, meeting the inclusion criteria totally and contained sufficient information on prevalence estimates. Of the nine articles excluded, three focused on adult caregivers and did not provide sufficient information for the calculation of the prevalence of young caregivers [28,35,36]. One reported data that were sourced from the Australian Bureau of Statistics (ABS), which utilised data obtained from the 2006 census, outside the study period [42,43]. Another study also used census data collected from 1996 to 2006 [47]. The rest provided data on young caregivers within the 15–19 years of age group. Thus, data on those between 15 and 17 years old could not be obtained from the results presented [41,46]. As a result, a total of six articles or reports were reviewed systematically [37,38,39,40,44,45]. From each of these studies, the prevalence estimates were extracted from the articles or reports by one researcher and verified by a second researcher. Figure 1 depicts the PRISMA flow chart for article search and selection.

In terms of study design, all reviewed studies used a cross-sectional design with a questionnaire survey, except one [39]. The Ireland study reported national census data. Most of these studies recruited a large sample ranging from 1281 to 653,219. One study included a sample of 1281 students aged 10–14 years; however, the sample was drawn purposively from only two schools in the state of Florida, USA [37]. As the purposive sample of the study was not representative of the young people population in Florida, USA, the selection bias was significant. As a result, the prevalence provided by this study was excluded from further exploration of the overall prevalence estimate of caregiving in young people. The second cross-sectional survey in Northern Ireland involved a total of 4192 schoolchildren from 292 primary schools, representing 32% of the total primary schools in Northern Ireland [45]. The third study from Austria included a population sample of 7403 students aged 10–14 years randomly selected from 85 schools and 474 classes from a region [40]. The most recent survey study by Leu recruited nearly 4000 students in 230 schools in Switzerland [44].

In terms of the sampling technique, most of the included studies utilised a relatively proper approach, except the one conducted in the USA. For example, the Australian report on a survey of disabilities and chronic diseases utilised a multistage random sampling technique for recruitment [38]. The sample should be considered representative of the youth population, with a high response rate of 80% [38]. Nagl-Cupal et al. employed multilevel probability sampling based on the proportions of school types in each area [40]. The data were collected in the Switzerland study using a two-stage stratified sampling technique [44]. For the quality of the included studies, most were well designed and had reasonably good survey methodologies, except the USA study, which included only two schools in Florida, resulting in a high risk of sampling bias. All studies used self-report questionnaires to determine the prevalence of caregiving. However, the methods of caregiving assessment were, overall, unrefined and imprecise. In four studies, the caregiving information was obtained by answering simple questions [37,38,40,45]. Only the study by Leu et al. utilised a validated instrument for assessing caregiving status. The details on the information extracted from these studies and reports are tabulated in Table 1.

The results of the prevalence estimates of caregiving in young people are summarised in Table 2, excluding the study by Cohen et al. [37] for the reason mentioned above. As shown, the overall prevalence estimates of caregiving by young people under 18 years of age could not be obtained or calculated from the included studies because the age group cut-offs in these studies were <15 and 15–19 years. As such, the prevalence for the age between 15 and 17 years could not be calculated from the available information provided in the articles. However, the prevalence of caregiving among young people < 15 years was provided or could be calculated from the available information, resulting in a range between 1.1% (1.06–1.14%) in the Ireland study and 12.0% (11.02–12.98%) in the Northern Ireland study. Leu et al. provided an estimate of 7.9% (95%C.I. 6.7–9.3%) of caregiving among children aged 10–15 years old [44].

## 4. Discussion and Conclusions

Given the growing demand to care for patients with disabilities and chronic diseases, as well as the aging population in recent years, informal and unpaid caregiving provided by family members or relatives is also increasing. As noted, caregiving is not only confined to adults; many young people also provide care to others. (2) This phenomenon will likely continue. Given the possible negative impacts of caregiving on the growth and development of young people, particularly children and young adolescents, there is an urgent need to examine the extent of caregiving in young people globally [18,19,20,21,22,23,24]. This motivated the current systematic review, aiming to gain a better understanding of the magnitude of caregiving in young people. The results suggest that a proportion of young people assume a caregiver’s role and provide daily unpaid care to a family member or relative globally. The prevalence of caregiving ranged from about 1% to 12% in younger adolescents aged <15 years, and one study reported 8% in an age group between 10 and 15 years. These results reflect the imprecision of the estimate and the intrinsic difficulties of these studies. As there is no previous systematic review on the prevalence of caregiving among young people in the literature, a comparison of results would be difficult.

The results obtained from this systematic review have important implications in many areas, including child and adolescent development, physical and mental health, education, and future employment. The results suggest that as many as 12% of adolescents aged <15 years are involved in providing care to a family member or relatives; this represents a large number of young people when translated into the actual population size. As noted, over-burdening with a caregiving role in young people during the foundation period of their growth will affect the ongoing developmental trajectory, resulting in long-term sequela [18,19,20,21,22,23,24]. These long-term effects are affecting not just the individuals and their immediate families but also the coming generation as well as the economics and productivity of society. Given the significance of these effects on individuals and communities, as well as the possible increase in the need for unpaid caregiving across all age groups due to the increasing prevalence of chronic diseases globally, the policy implications are evident.

This current review has strengths and weaknesses. For its strengths, the structured PRISMA guidelines for systematic review were followed. The guidelines were established to ensure that proper and systematic procedures are implemented in the process of systematic reviews. To ensure the quality of this review, the guidelines were followed thoroughly, with members of the team reviewing the full process, including the selection and inclusion of articles and reports independently. Secondly, of the six included studies, two provided population data with a comprehensive and well-designed data collection mechanism. This ensured that the data were of excellent quality for review. Some limitations were also identified. Firstly, a range of operating definitions of caregiving were used in these studies. Moreover, in the majority of these studies, a single question was used to elicit information on caregiving and to determine the carer’s status. Only one study utilised a standardised and validated instrument to assess caregiving activities. Such a lack of uniformity in the operating definition, compounded with the inadequate assessment of the caregiving activities provided by the children, constitutes a severe shortcoming in these studies. The deficiency in the scientific rigour of the research methodologies, in turn, affects the prevalence estimate reported in these studies. This could be one of the reasons for such a wide range of estimates, ranging from 1% to 12%, reflected in these studies. Secondly, there was a lack of information on the 15–17 years of age group. As such, the overall prevalence of young caregivers aged under 18 years could not be estimated. Thirdly, studies included were mainly conducted in developed countries, including Australia, Ireland, and Austria; none had been found in developing or underdeveloped countries, such as countries in the Asia-Pacific region, South America, or Africa. Two possibilities explain the lack of studies from these regions: (1) there is a lack of awareness of the issue of caregiving among young people, or, (2) culturally, caregiving has been accepted as a norm for young family members to provide care to relatives within the family. It is worth noting that cultural norms and ethnic background may be important factors influencing the disclosure of any involvement in active caregiving activities, resulting in an underestimation of the burden of caregiving on young people. Furthermore, the normative acceptance of caregiving duties in young people in some communities may have, unknowingly, created a barrier for young people in seeking support when they feel the caregiving duties are too much of a burden to carry. Inadvertently, this may also render these young people to becoming a “hidden” population. These limitations in this current research call for an urgent need to examine the magnitude of caregiving in young people globally.

In conclusion, the prevalence of caregiving among young people was estimated to be as low as 1% but could be as high as 12% in some geographic regions. This figure only reflects the magnitude of caregiving in adolescents in developed countries. For other regions of the world, the burden of caregiving on young people is, by and large, unknown, calling for urgent action to further investigate the prevalence of caregiving in developing and underdeveloped countries.

## Figures and Tables

**Table 1:** Information extracted from individual studies on the prevalence of young carers.

Author, Year, Place	Age Groups Included	Type of Report	Information Source/Study Methods	Population Size	Results	Comments
Cohen et al., 2012, USA [37]	10–14 years	Journal article	Information was obtained from a cross-sectional survey administered to 1281 students from two schools in Florida. Students responded to questions asking whether they lived with the person who required care from the respondent and the type of assistance provided by the caregiver.	N = 1281 of a sample obtained from schools	459 (35.8%) reported as a caregiver, with 249 (19.4%) boys and 210 (16.4%) girls. Information on the extent, type, and duration of caregiving was not available.	Only two schools that did not constitute a representative sample were included in the study. This was a purposive sample targeting schools with a high prevalence of young caregivers. This constitutes selection bias.
Central Statistics Office, 2012, Ireland [39]	All ages, including 0–17 years	Government report on national census data	Census data based on standard data collection procedures.	N = 653,219, with 370,200 aged 14 years or younger and 283,019 aged between 15 and 19 years.	In total, 8472 (1.3%) young caregivers were identified among 4228 (1.1%) children and young adolescents aged <15 years, as well as 4244 (1.5%) of young caregivers aged 15–19 years. Information on the extent, the types, and the duration of caregiving was unavailable.	Population data with direct calculation of the prevalence
Nagl-Cupal et al., 2014, Austria [40]	10–14 years	Journal article	Data were collected from a cross-sectional survey of 85 schools and 474 classes, 4 school grades between 5th and 8th grade from 2 populous provinces in Austria. Multilevel probability samples based on the proportions of school types in each area were generated. A young caregiver was defined as one who provided care for patients with long-term illness or disability.	In total, 7403 responded to the survey with useful information.	335 (4.5%) young caregivers identified with 234 girls and 101 boys. For the extent of caregiving, 81% of them helped their mother ‘often’ or ‘very often’; 63% and 60% reported helping a sibling and their father. No information on the duration was available.	A well-designed study with a random and representative sample. The only drawback was a low response rate of 47.2%.
Australian Bureau of Statistics, 2015, Australia [38]	All ages, including 0–19 years; the age groups were categorised as <15 years and 15–24 years	Government report on national survey data	A survey of disabilities and chronic diseases was conducted using a multistage sampling technique. Information on caring status was elicited through questions on care provision.	25,555 responded to the survey with useful information on caregiving. However, there was no information on the number of respondents broken down by age group.	1.3% of young people aged <15 years were primary caregivers. Information on the extent, type, and duration of caregiving was not available.	The survey achieved a high response rate of 80% with little selection biases. However, due to the lack of information on the number of respondents by age group, further calculation on the prevalence was difficult.
Llyod, 2013, Northern Ireland [45]	10 and 11 years	Journal article	Information was collected through an online survey of 292 primary schools, representing 32% of the total 899 primary schools in Northern Ireland. Caregiving was identified as a positive answer to a question asking whether the child helped looking after someone in the family.	A total of 4192 children participated in the survey, representing 50% of the participating schools.	12% of the respondents reported having provided care to someone living with them. The types of care provided were mostly physical personal care, such as getting out of bed, walking, and dressing. No information on the duration of caregiving was reported.	A study with a large sample. However, it was not certain whether the sample was generated randomly. According to the description in the Methods, it was unlikely a random sample.
Leu et al., 2019, Switzerland [44]	10–15 years	Journal article	Data were collected from a cross-sectional survey conducted in 230 schools in Switzerland using a 2-stage stratified sampling approach. The caregiving status was assessed using them Multidimensional Assessment of Caring Activities (MACA-YC18).	A total of 3991 students responded and provided useful data.	7.9% (95%C.I. 6.7–9.3%) of children responded positively to the question on caring for a family member. In terms of the extent of caregiving, 17% and 22% reported providing very high and high amounts of caring, respectively; 32% and 30% provided a moderate and low amount of care. This involved emotional, financial, and personal care. There was quantifiable information on the duration of caregiving.	A high response rate of 97.8% was achieved with reasonably little selection bias. The prevalence estimated was also weighed to adjust for the sampling effect.

**Table 2:** The estimated prevalence of caregiving in young people by study and age group.

Study	Prevalence (95%C.I.)	
	<15 Years	10–15 Years
Central Statistics Office Ireland [39]	1.1% (1.06–1.14%)	-
Llyod [45]	12.0% (11.02–12.98%)	-
Nagl-Cupal et al. [40]	4.5% (4.03–4.97%)	-
Australian Bureau of Statistics [38]	1.3% (1.16–1.44%)	-
Leu et al. [44]	-	7.9% (6.7–9.3%)

## Data Availability

Data were presented in this paper.
